# Supplementary material for: Single-layer graphene membranes by crack-free transfer for gas mixture separation
Source: Nat Commun. 2018 Jul 6;9:2632. doi: 10.1038/s41467-018-04904-3 (PMC6035196; doi:10.1038/s41467-018-04904-3)
Supplement: Supplementary file 1 — Supplementary Information [file 41467_2018_4904_MOESM1_ESM.pdf]

Supplementary information

Single-layer graphene membranes by crack-free transfer for gas mixture separation

Shiqi Huang et al.

Supplementary Figures

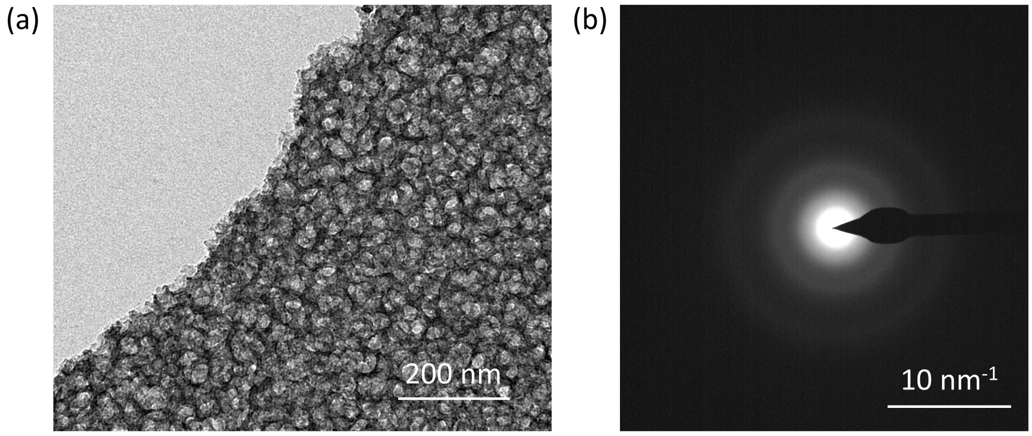

Supplementary Figure 1. Characterization of the standalone NPC film. a) Transmission electron microscopy (TEM) image and b) small area electron diffraction (SAED) of the standalone NPC film.

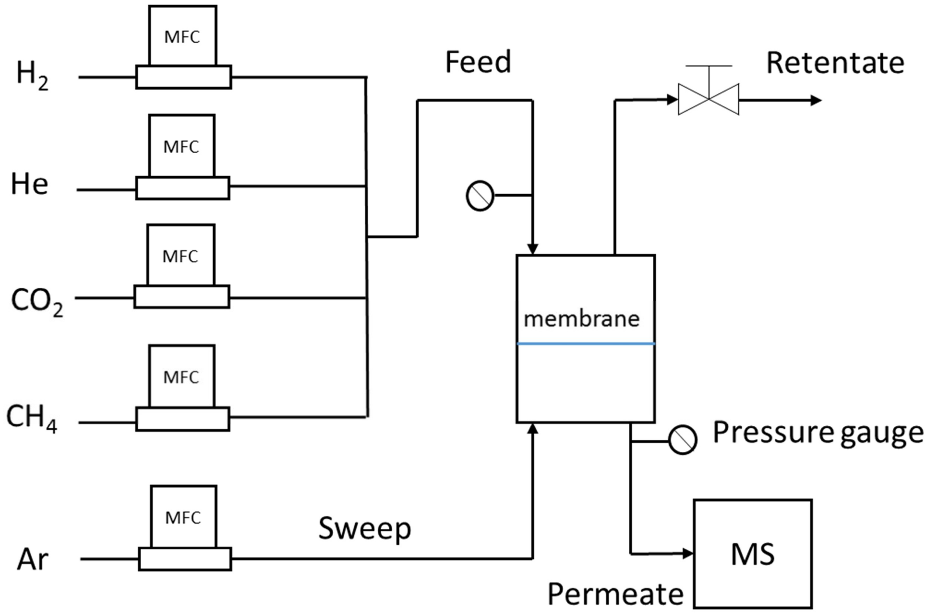

Supplementary Figure 2. Schematic of the setup for the gas permeance test.

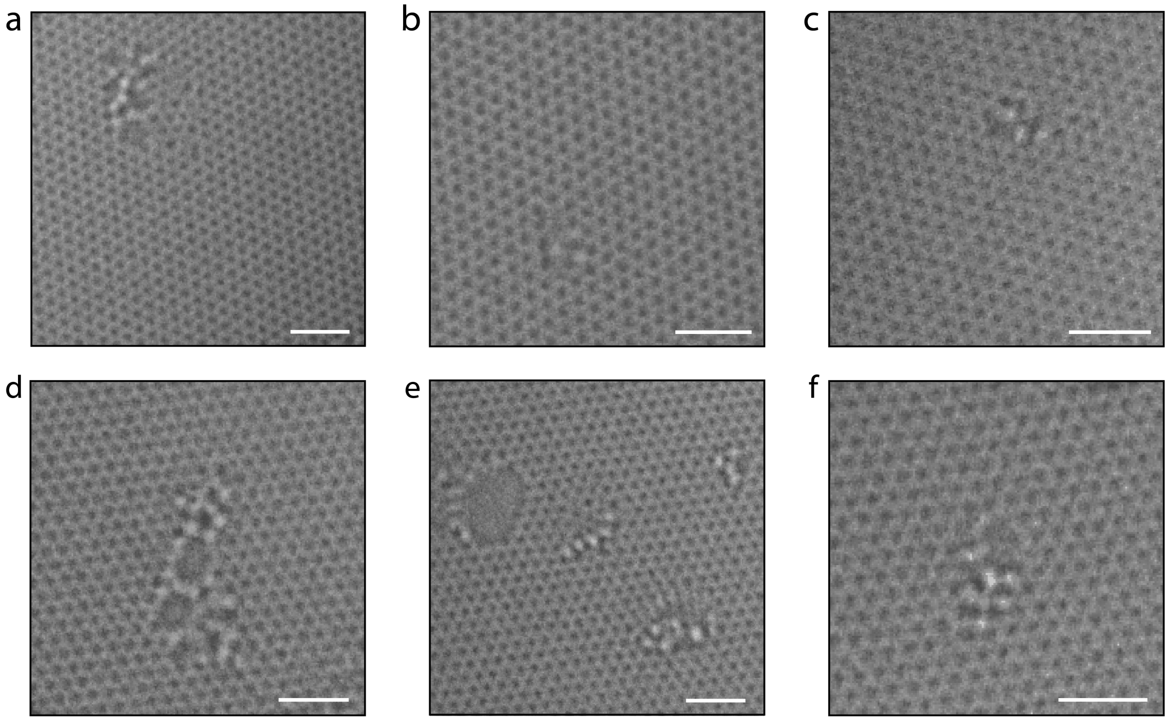

Supplementary Figure 3. Raw high-resolution transmission electron microscopy (HRETM) images of the nanopores in graphene. (a-c) Intrinsic defects in CVD graphene; (d-f) ozone-treated graphene (2 minutes at 80 ºC). All scale bars are 1 nm.

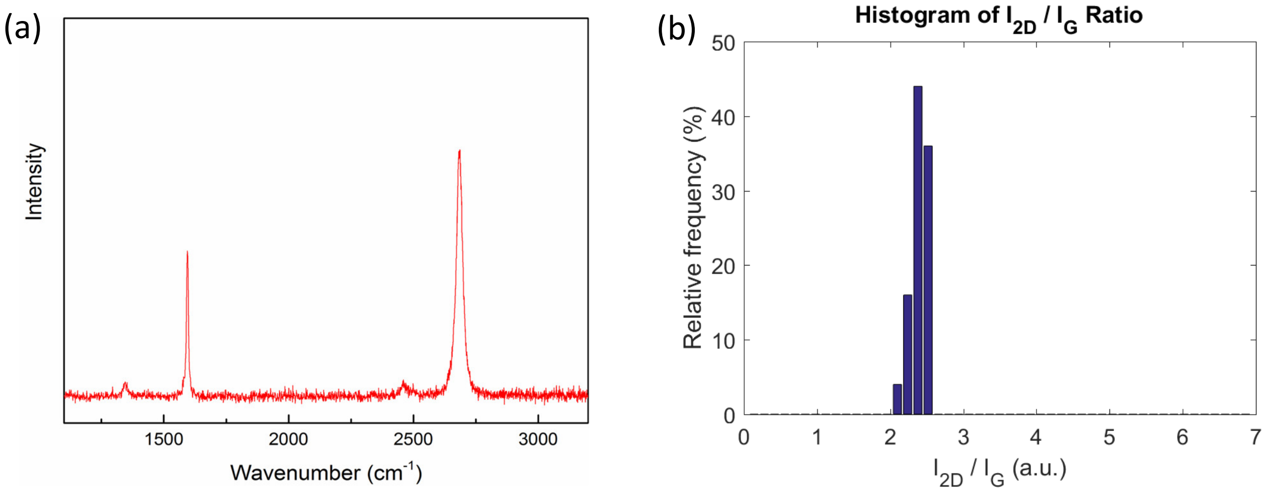

Supplementary Figure 4. a) Raman spectrum of the low pressure chemical vapor deposition (LPCVD) grown graphene. b) Histogram of I_{2D}/I_G from Raman mapping of LPCVD graphene.

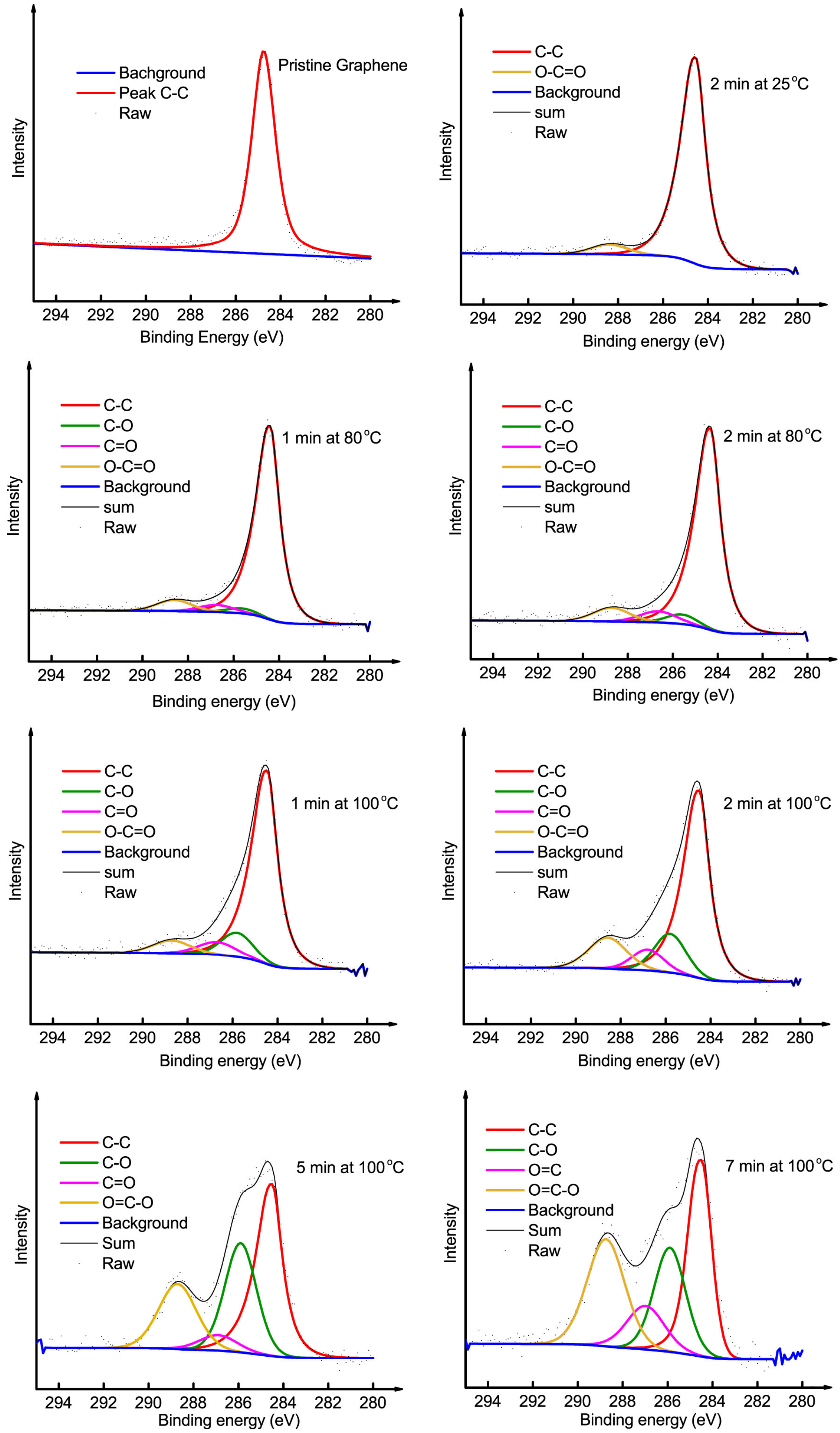

Supplementary Figure 5. X-ray photoelectron spectroscopy (XPS) spectra of functionalized graphene with different ozone treatments

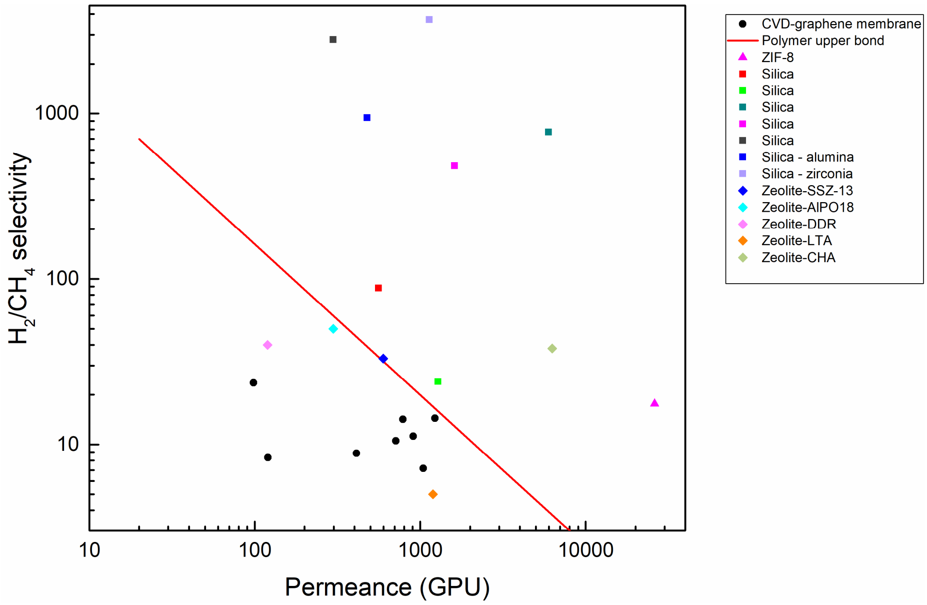

Supplementary Figure 6. A comparison of single-layer graphene films (all eight membranes) with a low-density of intrinsic defects (0.025%) with membranes in the literature in terms of the H_2/CH_4 separation performances. The red line is the polymer upper bond assuming 1 μm-thick skin layer[1], ZIF-8[2], silica[3–9] and zeolite membranes (SSZ-13[10], AlPO-18[11], DDR[12], LTA[13], CHA[14]).

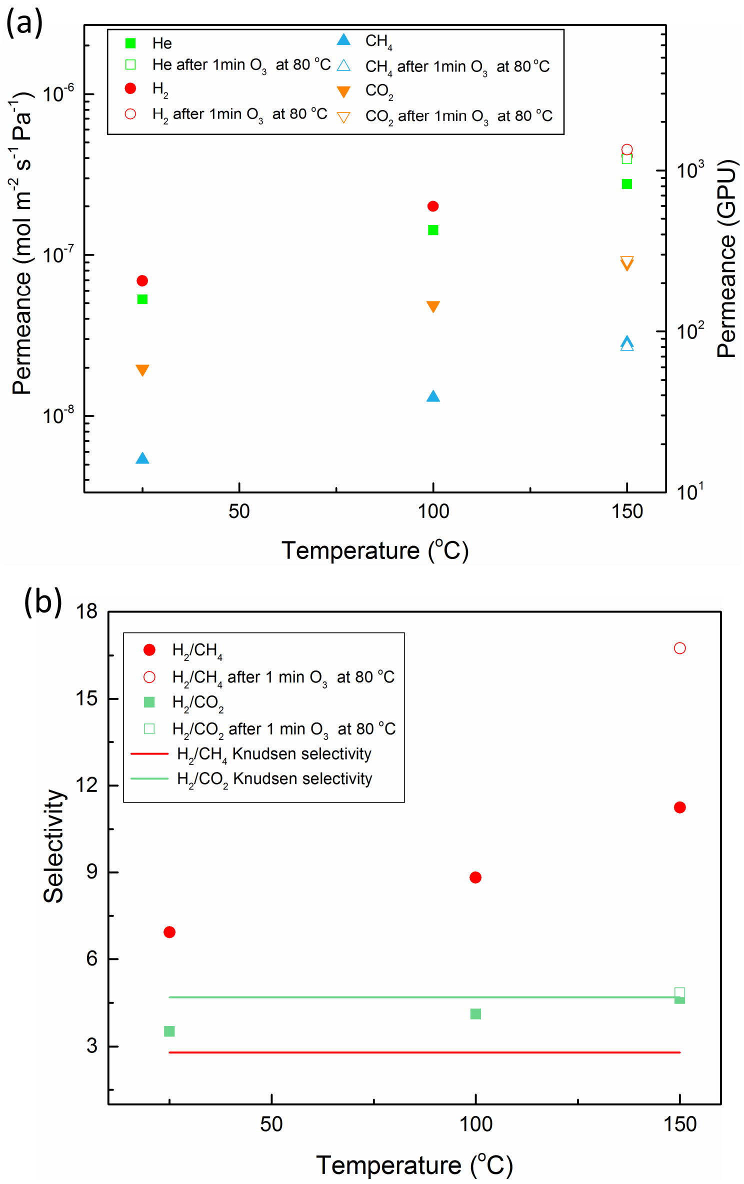

Supplementary Figure 7. Gas separation performance of membrane M5 treated by O_3 for 1 min at 80 ºC. a) gas permeances of H_2 and CH_4, and b) H_2/CH_4 and H_2/CO_2 selectivities.

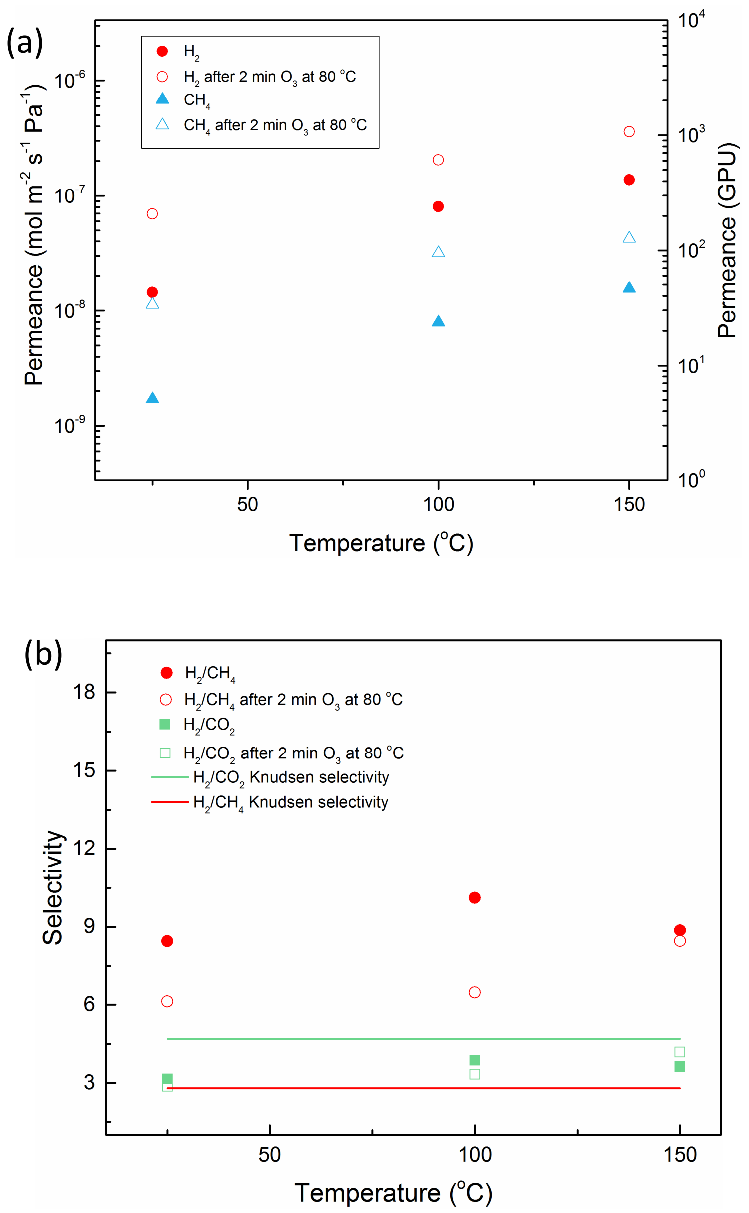

Supplementary Figure 8. Gas separation performance of M7 treated by 2 min O_3 at 80 ºC, a) gas permeance of H_2 and CH_4, b) gas selectivity between H_2/CH_4 and H_2/CO_2.

Supplementary Tables

Supplementary Table 1. Gas permeance through the standalone nanoporous carbon (NPC) film.

Gas	Permeance (mol m^{-2} s^{-1} Pa^{-1})
H_2	1.9 x10^{-3}
He	1.1 x10^{-3}
CH_4	7.8 x10^{-4}
N_2	6.0 x10^{-4}
CO_2	4.4 x10^{-4}

Supplementary Table 2. E_{act} for gas diffusion across the intrinsic defects in graphene.

E_{act} (kJ/mol)	He	H_2	CO_2	CH_4
M1	16.3	20.6	30.0	27.8
M2	15.3	20.5	29.8	24.4
M3	20.8	25.2	37.2	32.4
M4	11.4	17.6	29.6	22.7
M5	13.6	18.7	29.4	26.5
M6	11.8	17.1	30.7	24.8
M7	17.5	23.3	34.9	31.6
M8	10.8	18.2	29.1	16.5

Supplementary Table 3. $C_o A_{act} A_{sur}$ extracted by adsorption model fitting to the observed transport from the intrinsic defects of graphene membranes.

$C_o A_{act} A_{sur}$	He	H_2	CO_2	CH_4
M1	3.05 x 10^{-6}	4.70 x 10^{-6}	2.82 x 10^{-7}	3.52 x 10^{-7}
M2	1.51 x 10^{-5}	2.49 x 10^{-5}	1.85 x 10^{-6}	5.74 x 10^{-7}
M3	6.13 x 10^{-5}	9.14 x 10^{-5}	1.24 x 10^{-5}	3.73 x 10^{-6}
M4	4.55 x 10^{-6}	1.24 x10^{-5}	1.85 x 10^{-6}	3.74 x10^{-7}
M5	1.24 x 10^{-5}	2.49 x 10^{-5}	3.05 x10^{-6}	1.24 x 10^{-6}
M6	6.33 x 10^{-6}	1.37 x 10^{-5}	4.12 x 10^{-6}	1.37 x 10^{-6}
M7	1.24 x 10^{-5}	3.72 x 10^{-5}	6.79 x 10^{-6}	3.05 x 10^{-6}
M8	7.52 x 10^{-7}	1.76 x 10^{-6}	1.02 x 10^{-7}	4.59 x 10^{-9}

Supplementary Table 4. $E_{act-app}$ of membrane M2 before and after ozone functionalization (2 min at 25 ºC).

$E_{app-act}$ (kJ/mol)	He	H_2	CO_2	CH_4
Intrinsic defects	15.3	16.5	12.8	11.4
Post-functionalization	12.9	11.8	12.4	5.0

Supplementary Table 5. $C_o A_{act} A_{sur}$ of membrane M2 before and after ozone functionalization (2 min at 25 ºC).

$C_o A_{act} A_{sur}$	He	H_2	CO_2	CH_4
Intrinsic defects	1.5 x 10^{-5}	2.5 x 10^{-5}	1.9 x 10^{-6}	5.7 x 10^{-7}
Post-functionalization	4.1 x 10^{-6}	3.4 x 10^{-6}	6.8 x 10^{-7}	2.8 x10^{-8}

Supplementary Table 6. $E_{act-app}$ of membrane M8 before and after ozone functionalization (2 min at 100 ºC).

$E_{app-act}$ (kJ/mol)	He	H_2	CO_2	CH_4
Intrinsic defects	10.8	14.2	12.1	3.5
Post-functionalization	12.9	12.0	8.2	6.3

Supplementary Table 7. $C_o A_{act} A_{sur}$ of membrane M8 before and after ozone functionalization (2 min at 100 ºC).

$C_o A_{act} A_{sur}$	He	H_2	CO_2	CH_4
Intrinsic defects	$7.5\text{x}10^{-7}$	$1.8\text{x}10^{-6}$	$1.0\text{x}10^{-7}$	$4.6\text{x}10^{-9}$
Post-functionalization	$7.5\text{x}10^{-6}$	$4.5\text{x}10^{-6}$	$2.5\text{x}10^{-7}$	$5.6\ \text{x}10^{-8}$

Supplementary Table 8. The estimated density of large, non-selective pores in graphene as a function of α_{H_2/CH_4}.

α_{H_2/CH_4}	C_e/C_a	PPM of nanopores (with respect to all nanopores) with an electron density gap larger than 0.38 nm
5	$2.5 \text{ x } 10^{-5}$	25.0
10	$1.1\text{x } 10^{-5}$	11.1
15	$7.1 \text{ x } 10^{-6}$	7.1
20	$5.3 \text{ x } 10^{-6}$	5.3
25	$4.2 \text{ x } 10^{-6}$	4.2

Supplementary Table 9. He permeance* through the intrinsic defects of membranes M1-M8

Temperature (ºC)	M1	M2	M3	M4	M5	M6	M7	M8
25	4.0 x10^{-9}	3.0 x10^{-8}	1.5 x10^{-8}	4.8x10^{-8}	5.3 x10^{-8}	5.5 x10^{-8}	1.1 x10^{-8}	1.0 x10^{-8}
100	1.9 x10^{-8}	9.9 x10^{-8}	6.4 x10^{-8}	9.1 x10^{-8}	1.4 x10^{-7}	1.4 x10^{-7}	5.2 x10^{-8}	2.1 x10^{-8}
150	3.2 x10^{-8}	1.9 x10^{-7}	1.9 x10^{-7}	2.0 x10^{-7}	2.8 x10^{-7}	2.3 x10^{-7}	8.7 x10^{-8}	3.4 x10^{-8}
200	4.4 x10^{-8}							5.5 x10^{-8}

* Unit of permeance is mol m^{-2} s^{-1} Pa^{-1}

Supplementary Table 10. CO_2 permeance* through the intrinsic defects of membranes M1-M8

Temperature (ºC)	M1	M2	M3	M4	M5	M6	M7	M8
25	1.5 x10^{-9}	1.0 x10^{-8}	3.8 x10^{-9}	1.2 x10^{-8}	2.0 x10^{-8}	1.6 x10^{-8}	4.6 x10^{-9}	7.2 x10^{-10}
100	4.6 x10^{-9}	2.9 x10^{-8}	1.6 x10^{-8}	2.5 x10^{-8}	4.9 x10^{-8}	4.8 x10^{-8}	2.1 x10^{-8}	2.0 x10^{-9}
150	6.7 x10^{-9}	4.7 x10^{-8}	4.5 x10^{-8}	6.1 x10^{-8}	8.9 x10^{-8}	8.2 x10^{-8}	3.8 x10^{-8}	2.7 x10^{-9}
200	7.5 x10^{-9}							4.7 x10^{-9}

* Unit of permeance is mol m^{-2} s^{-1} Pa^{-1}

Supplementary Table 11. CH_4 permeance* through the intrinsic defects of membranes M1-M8

Temperature (ºC)	M1	M2	M3	M4	M5	M6	M7	M8
25	8.6 x10^{-10}	5.8 x10^{-9}	1.6 x10^{-9}	8.1 x10^{-9}	5.4 x10^{-9}	1.2 x10^{-8}	1.7 x10^{-9}	1.1 x10^{-9}
100	3.3 x10^{-9}	1.4 x10^{-8}	5.1 x10^{-9}	1.4 x10^{-8}	1.3 x10^{-8}	2.9 x10^{-8}	7.9 x10^{-9}	1.4 x10^{-9}
150	4.8 x10^{-9}	2.3 x10^{-8}	1.9 x10^{-8}	2.7 x10^{-8}	2.9 x10^{-8}	4.9 x10^{-8}	1.6 x10^{-8}	1.4 x10^{-9}
200	4.7 x10^{-9}							1.9 x10^{-9}

* Unit of permeance is mol m^{-2} s^{-1} Pa^{-1}

Supplementary Notes

Supplementary Note 1: Characterization of the NPC film

The standalone NPC film[15] was fabricated and characterized as the control. Briefly, the precursor polymer solution was coated on bare copper foil, pyrolyzed, and then transferred to a TEM grid and a porous tungsten substrate. Bright-field transmission electron microscopy (TEM) image and the small area electron diffraction (SAED) pattern revealed that the NPC film was amorphous (Supplementary Figure 1). Image analysis of the pore-size-distribution (PSD) in the NPC film revealed pores with diameter between 20-30 nm. Gas permeance test on the porous W supported NPC film exhibited gigantic H_2 permeance 1.9 x 10^{-3} mol m^{-2} s^{-1} Pa^{-1} (Supplementary Table 1)[15]. The high-permeance of the NPC film ensures that the NPC film does not restrict the gas permeance from the nanopores in the single-layer graphene.

Supplementary Note 2: Fabrication of the macroporous tungsten substrate

The macroporous substrate was fabricated by drilling an array of 5 μm holes (2500 holes) in 1 mm^2 area of a 50-μm-thick W foil. Laser drilling was carried out by Potomac Photonics Inc. A W foil was chosen as membrane support due to its rigidity, mechanical and thermal stability. More importantly, the W support enabled metal-to-metal sealing, which was gas-tight. Hydrogen permeances through these 5 μm holes exceeded 10^{-2} mol m^{-2} s^{-1} Pa^{-1}. Therefore, like NPC film, the W support also does not restrict the gas-transport through the single-layer graphene membrane.

Supplementary Note 3: Gas transport model

The adsorbed phase transport of gases through pores on the graphene can be summarized in following steps.[16–18]

Single-component feed

1) Gas adsorption on the graphene lattice;

$$l + A \overset{k_1}{\Leftrightarrow} A_l \tag{1}$$

Adsorption rate:

$$k_1 P_A[l] - k_{-1}[A_l] \tag{2}$$

2) 2D diffusion of adsorbed gas towards the pores on graphene;

$$A_l + B_l \overset{k_2}{\Leftrightarrow} AB_l + l \tag{3}$$

Pore-association rate:

$$k_2[A_l][B_l] - k_{-2}[AB_l][l] \tag{4}$$

3) Gas diffusion across the pore;

$$AB_l \overset{k_3}{\Leftrightarrow} RB_m \tag{5}$$

Diffusion rate:

$$k_3[AB_l]-k_{-3}[RB_m] \tag{6}$$

4) Dissociation of the molecule from the pore and 2D diffusion of the molecule away from the pore (permeate side).

$$RB_m + m \overset{k_4 = k_{-2}}{\Longleftrightarrow} R_m + B_m \tag{7}$$

Dissociation rate:

$$k_4[RB_m][m] - k_{-4}[R_m][B_m] \tag{8}$$

5) Desorption of molecular from the graphene lattice (permeate side).

$$R_m \overset{k_5 = k_{-1}}{\Longleftrightarrow} R + m \tag{9}$$

Desorption rate:

$$k_5[R_m] - k_{-5} P_R[m] \tag{10}$$

A: Feed gases with P_A; R: permeate gases with P_R;
l: Available adsorption sites of the graphene lattice (feed side);
m: Available adsorption sites of the graphene lattice (permeate side);
A_l: Gas adsorbed on graphene (feed side);
R_m: Gas adsorbed on graphene (permeate side);
B_l: Empty holes on graphene (feed side);
B_m: Empty holes on graphene (permeate side);
AB_l: Gas associated with hole (feed side);
RB_m: Gas associated with hole (permeate side);
k_i: rate constant for step i;
k_{-i}: backward rate constant for step i.

Typically, the rate-limiting step is the diffusion step (step 3), while all other steps can be considered at the equilibrium.

Adsorption equilibrium:

$$K_1 = \frac{k_1}{k_{-1}} = \frac{[A_l]}{P_A[l]} \tag{11}$$

Pore-association equilibrium:

$$K_2 = \frac{k_2}{k_{-2}} = \frac{[AB_l][l]}{[A_l][B_l]} \tag{12}$$

Pore-dissociation rate:

$$K_4 = \frac{k_4}{k_{-4}} = \frac{[R_m][B_m]}{[RB_m][m]} \tag{13}$$

Desorption rate:

$$K_5 = \frac{k_5}{k_{-5}} = \frac{P_R[m]}{[R_m]} \tag{14}$$

Total sites of adsorption sites can be fixed as C_l,

$$C_l = [A_l] + [l] = [m] + [R_m] \tag{15}$$

Langmuir adsorption was assumed to describe the adsorption of gases on the graphene lattice; fraction of sites occupancy could be expressed as $\theta_A = \frac{K_1 P_A}{1+K_1 P_A}$.

$$[A_l] = \frac{C_l K_1 P_A}{1+K_1 P_A} \tag{16}$$

$$[l] = \frac{C_l}{1+K_1 P_A} \tag{17}$$

Assuming that the total pore sites is fixed as C_o

$$C_o = [AB_l] + [B_l] = [B_m] + [RB_m] \tag{18}$$

$$[AB_l] = \frac{C_0 K_1 K_2 P_A}{1+K_1 K_2 P_A} \tag{19}$$

Overall transport rate = rate of molecular diffusion through the pores

$$k_3[AB_l] - k_{-3}[RB_m] = C_o \frac{k_1}{k_{-1}} \frac{k_2}{k_{-2}} k_3 \left(\frac{p_A}{1+\frac{k_1}{k_{-1}}\frac{k_2}{k_{-2}} p_A} - \frac{p_R}{1+\frac{k_1}{k_{-1}}\frac{k_2}{k_{-2}} p_R}\right) \tag{20}$$

From classical transition state theory,

$$\frac{k_1}{k_{-1}}\frac{k_2}{k_{-2}} = A_{sur}exp(-\frac{\Delta E_{sur}}{RT}) \quad (21)$$

$$k_3 = A_{act}exp(-\frac{E_{act}}{RT}) \quad (22)$$

$$Flux = C_o A_{act} A_{sur} exp(-\frac{E_{act}+\Delta E_{sur}}{RT})(\mathrm{f}(P_A) - \mathrm{f}(P_R)) \quad (23)$$

$$* \ \mathrm{f}(P_x) = \frac{P_x}{1+A_{sur}exp(-\frac{\Delta E_{sur}}{RT})P_x}$$

where A_{act} and A_{sur} are the pre-exponential factors, E_{act} is the activation energy to diffuse across the pores, and E_{sur} is the energy of adsorption on the graphene pore.

Mixture feed

The key steps in the transport of gases in the mixture feed can be written as following (for the component i)

1) Gas adsorption on the graphene lattice;

$$l + A_i \overset{k_1}{\Leftrightarrow} A_{i,l} \quad (24)$$

Adsorption rate:

$$k_1 P_{i,A}[l] - k_{-1}[A_{i,l}] \quad (25)$$

2) 2D diffusion of adsorbed gas towards the pores on graphene;

$$A_{i,l} + B_l \overset{k_2}{\Leftrightarrow} A_i B_l + l \quad (26)$$

Pore-association rate:

$$k_2[A_{i,l}][B_l] - k_{-2}[A_i B_l][l] \quad (27)$$

3) Gas diffusion across the pore;

$$A_i B_l \overset{k_3}{\Leftrightarrow} R_i B_m \quad (28)$$

Diffusion rate:

$$k_3[A_i B_l] - k_{-3}[R_i B_m] \quad (29)$$

4) Dissociation of the molecule from the pore and 2D diffusion of the molecule away from the pore (permeate side).

$$R_i B_m + m \overset{k_4 = k_{-2}}{\Longleftrightarrow} R_{i,m} B_m \quad (30)$$

Dissociation rate:

$$k_4[R_i B_m][m] - k_{-4}[R_{i,m}][B_m] \quad (31)$$

5) Desorption of molecular from the graphene lattice (permeate side).

$$R_{i,m} \overset{k_5 = k_{-1}}{\Longleftrightarrow} R_i + m \quad (32)$$

Desorption rate:

$$k_5[R_{i,m}] - k_{-5}\, P_{i,R}[m] \tag{33}$$

Total sites of adsorption sites can be fixed as C_l,

$$C_l = \sum[A_{i,l}] + [l] = [m] + \sum[R_{i,m}] \tag{34}$$

Assumed total pore sites is fixed as C_o

$$C_o = \sum[A_i B_l] + [B_l] = [B_m] + \sum[R_i B_m] \tag{35}$$

Therefore, combining Eq. S25-S35, one can arrive to

$$\mathrm{Flux}_i \ = \ C_o\, A_{act,i}\, A_{sur,i}\, exp\left(-\frac{(E_{\mathrm{act,i}} + \Delta E_{sur,i})}{RT}\right)\left(g\left(P_{i,A}\right) - g\left(P_{i,R}\right)\right) \tag{36}$$

$$*\ g(P_x) = \frac{P_x}{1 + \sum_1^n A_{sur}\, exp\left(\frac{-\Delta E_{sur}}{RT}\right) P_x}$$

The transport model was fitted to the temperature dependent experimental permeation data to obtain apparent activation energy $E_{app-act} = E_{act} + E_{sur}$ and products of pore density and pre-exponential factors $C_o A_{act} A_{sur}$. Based on the literature,[19] $E_{CO2,sur}$= -17 kJ/mol, $E_{CH4,sur}$= -13 kJ/mol, $E_{H2,sur}$= -4 kJ/mol, $E_{He,sur}$= 0 kJ/mol, the E_{act} could be calculated by the equation $E_{act} = E_{act-app} - E_{sur}$.

Supplementary Note 4: Estimation of the density of the non-selective nanopores in graphene

Given that the transmission coefficient for H_2 and CH_4 from a large nanopore (effusive transport, ca. 10^{-18} mol s^{-1} Pa^{-1}) is several orders of magnitude higher than that from a molecular-selective pore (activated transport, 10^{-22} - 10^{-23} mol s^{-1} Pa^{-1} for H_2, and 10^{-26} mol s^{-1} Pa^{-1} for CH_4),[20–23] we expect that the percentage of pores hosting an electron-density-gap larger than the size of CH_4 (kinetic diameter of 0.38 nm) in our graphene membrane is quite low, otherwise H_2/CH_4 selectivities greater than 2.8 could not be achieved.

Typically, the H_2 and CH_4 permeances from the graphene membrane of can be written as

$$Permeance_{H_2} = C_e\, N_{e,H_2} + C_a\, N_{a,H_2} \qquad (37)$$

$$Permeance_{CH_4} = C_e\, N_{e,CH_4} + C_a\, N_{a,CH_4} \qquad (38)$$

where $N_{e,H_2}$and $N_{e,CH4}$ correspond to the permeation coefficient of H_2 and CH_4, respectively for the effusive transport. N_{a,H_2} and N_{a,CH_4} correspond to the permeation coefficient of H_2 and CH_4, respectively for the activated transport. C_e and C_a correspond to the pore-density (number of pores per unit area) of pores corresponding to the effusive and the activated transport, respectively. The selectivity of gas, α_{H_2/CH_4}, is simply the ratio of the two permeances.

$$\alpha_{H2/CH4} = \frac{Permeance_{H_2}}{Permeance_{CH_4}} = \frac{C_e\, N_{e,H_2} + C_a\, N_{a,H_2}}{C_e\, N_{e,CH_4} + C_a\, N_{a,CH_4}} \qquad (39)$$

Rearranging above, we get

$$\alpha_{H_2/CH_4} = \left(\frac{N_{a,H_2}}{N_{a,CH_4}}\right)\left(\frac{1 + \frac{C_e}{C_a}\frac{N_{e,H_2}}{N_{a,H_2}}}{1 + \frac{C_e}{C_a}\frac{N_{e,CH_4}}{N_{a,CH_4}}}\right) \qquad (40)$$

Taking N_{a,H_2} and $N_{a,CH_4}$to be 10^{-22} and 10^{-26} mol s^{-1} Pa^{-1}, respectively,[21] and $N_{e,H_2}$and $N_{e,CH_4}$to be 10^{-18} mol s^{-1} Pa^{-1},[22] we get

$$\alpha_{H_2/CH_4} = 10^4 \left(\frac{1 + 10^4\frac{C_e}{C_a}}{1 + 10^8\frac{C_e}{C_a}}\right) \qquad (41)$$

Rearranging, we get

$$\frac{C_e}{C_a} = 10^{-4}\left(\frac{1 - 10^{-4}\alpha_{H_2/CH_4}}{\alpha_{H_2/CH_4} - 1}\right) \qquad (42)$$

Based on above, we list the percentage of large pores in the CVD graphene (Supplementary Table 8).

Supplementary Note 5: Statistics summary on the pores characterization by HRTEM

Statistics summary on the pores characterized by High-resolution transmission electron microscopy (HRTEM) were based on the pores located at a minimum distance of 2 nm away from the PMMA contaminations. 31 sub-1-nm nanopores and 10 bigger pores (1-5 nm) were found in the CVD graphene (survey area of 14200 nm^2), corresponding to a pore-density of 2.8 x 10^{11} cm^{-2}. We found 67 sub-1-nm nanopores and 10 bigger pores (1-5 nm) in the ozone-treated graphene in (survey area of 18200 nm^2), corresponding to a pore-density of 4.2 x 10^{11} cm^{-2}.

Supplementary References

1. Robeson, L. M. The upper bound revisited. *J. Memb. Sci.* **320,** 390–400 (2008).

2. He, G., Dakhchoune, M., Zhao, J., Huang, S. & Agrawal, K. V. Electrophoretic Nuclei Assembly for Crystallization of High Performance Membranes on Unmodified Supports. *Adv. Funct. Mater.* **(in press),** doi: 10.1002/adfm.201707427 (2018).

3. Castricum, H. L., Qureshi, H. F., Nijmeijer, A. & Winnubst, L. Hybrid silica membranes with enhanced hydrogen and CO_2 separation properties. *J. Memb. Sci.* **488,** 121–128 (2015).

4. Qureshi, H. F., Nijmeijer, A. & Winnubst, L. Influence of sol-gel process parameters on the micro-structure and performance of hybrid silica membranes. *J. Memb. Sci.* **446,** 19–25 (2013).

5. Vos, R. M. De & Verweij, H. High-Selectivity , High-Flux Silica Membranes for Gas Separation. **279,** 1710–1712 (1998).

6. Ahn, S. J., Yun, G. N., Takagaki, A., Kikuchi, R. & Oyama, S. T. Synthesis and characterization of hydrogen selective silica membranes prepared by chemical vapor deposition of vinyltriethoxysilane. *J. Memb. Sci.* **550,** 1–8 (2018).

7. Lee, D., Zhang, L., Oyama, S. T., Niu, S. & Saraf, R. F. Synthesis, characterization, and gas permeation properties of a hydrogen permeable silica membrane supported on porous alumina. *J. Memb. Sci.* **231,** 117–126 (2004).

8. Gu, Y., Hacarlioglu, P. & Oyama, S. T. Hydrothermally stable silica-alumina composite membranes for hydrogen separation. *J. Memb. Sci.* **310,** 28–37 (2008).

9. Ahn, S. J., Takagaki, A., Sugawara, T., Kikuchi, R. & Oyama, S. T. Permeation properties of silica-zirconia composite membranes supported on porous alumina substrates. *J. Memb. Sci.* **526,** 409–416 (2017).

10. Kosinov, N., Auffret, C., Borghuis, G. J., Sripathi, V. G. P. & Hensen, E. J. M. Influence of the Si/Al ratio on the separation properties of SSZ-13 zeolite membranes. *J. Memb. Sci.* **484,** 140–145 (2015).

11. Wang, B., Hu, N., Wang, H., Zheng, Y. & Zhou, R. Improved AlPO-18 membranes for light gas separation. *J. Mater. Chem. A* **3,** 12205–12212 (2015).

12. Himeno, S. *et al.* Synthesis and permeation properties of a DDR-type zeolite membrane for separation of CO_2/CH_4 gaseous mixtures. *Ind. Eng. Chem. Res.* **46,** 6989–6997 (2007).

13. Huang, A., Wang, N. & Caro, J. Synthesis of multi-layer zeolite LTA membranes with enhanced gas separation performance by using 3-aminopropyltriethoxysilane as interlayer. *Microporous Mesoporous Mater.* **164,** 294–301 (2012).

14. Kida, K., Maeta, Y. & Yogo, K. Pure silica CHA-type zeolite membranes for dry and humidified CO_2/CH_4mixtures separation. *Sep. Purif. Technol.* **197,** 116–121 (2018).

15. K. V. Agrawal, M. Dakachoune, S. Huang, G. He, N. D. 'Ultrahigh Flux Gas -selective Nanoporous Carbon Membrane and Manufacturing Method Thereof',. *Application PCT/EP2017/057684.*

16. Lee W. Drahushuk and Michael S. Strano. Mechanisms of Gas Permeation through Single Layer Graphene Membranes. *Langmuir* **28,** 16671–16678 (2012).

17. de Lange, R. S. A., Keizer, K. & Burggraaf, A. J. Analysis and Theory of Gas Transport in Micropouros Sol-Gel Derived Ceramic Membranes. *J. Memb. Sci.* **104,** 81–100 (1995).

18. Barrer, R. M. Porous crystal membranes. *J. Chem. Soc. Faraday Trans.* **86,** 1123–1130 (1990).

19. Schrier, J. Fluorinated and nanoporous graphene materials as sorbents for gas separations. *ACS*

Appl. Mater. Interfaces **3,** 4451–4458 (2011).

20. Wang, L. *et al.* Fundamental transport mechanisms, fabrication and potential applications of nanoporous atomically thin membranes. *Nat. Nanotechnol.* **12,** 509–522 (2017).

21. Koenig, S. P., Wang, L., Pellegrino, J. & Bunch, J. S. Selective molecular sieving through porous graphene. *Nat. Nanotechnol.* **7,** 728–32 (2012).

22. Celebi, K. *et al.* Ultimate Permeation Across Atomically Thin Porous Graphene. *Science.* **344,** 289–292 (2014).

23. Yuan, Z. *et al.* Mechanism and Prediction of Gas Permeation through Sub-Nanometer Graphene Pores: Comparison of Theory and Simulation. *ACS Nano* **11,** 7974–7987 (2017).
